# Drought in the West Indes

**Published:** 1864-02

**Authors:** 


Drought in the West Indies.—A drought of more than twelve months' duration in the West Indies has caused much suffering and disease to the inhabitants; no rain having fallen in Guadaloupe and other islands of the Antilles since August 16th, 1862. The mortality among the white population has been very great.

